# Circ-LocNet: A Computational Framework for Circular RNA Sub-Cellular Localization Prediction

**DOI:** 10.3390/ijms23158221

**Published:** 2022-07-26

**Authors:** Muhammad Nabeel Asim, Muhammad Ali Ibrahim, Muhammad Imran Malik, Andreas Dengel, Sheraz Ahmed

**Affiliations:** 1 German Research Center for Artificial Intelligence (DFKI), 67663 Kaiserslautern, Germany; muhammad_ali.ibrahim@dfki.de (M.A.I.); andreas.dengel@dfki.de (A.D.); sheraz.ahmed@dfki.de (S.A.); 2 Department of Computer Science, Technical University of Kaiserslautern, 67663 Kaiserslautern, Germany; 3 School of Computer Science & Electrical Engineering, National University of Sciences and Technology, Islamabad 44000, Pakistan; malik.imran@seecs.edu.pk; 4 DeepReader GmbH, Trippstadter Str. 122, 67663 Kaiserslautern, Germany

**Keywords:** circular RNA, non-coding RNA, subcellular localization, machine learning, nucleotide frequency, nucleotide physico-chemical properties, classification, web server, sub-cellular localization dataset

## Abstract

Circular ribonucleic acids (circRNAs) are novel non-coding RNAs that emanate from alternative splicing of precursor mRNA in reversed order across exons. Despite the abundant presence of circRNAs in human genes and their involvement in diverse physiological processes, the functionality of most circRNAs remains a mystery. Like other non-coding RNAs, sub-cellular localization knowledge of circRNAs has the aptitude to demystify the influence of circRNAs on protein synthesis, degradation, destination, their association with different diseases, and potential for drug development. To date, wet experimental approaches are being used to detect sub-cellular locations of circular RNAs. These approaches help to elucidate the role of circRNAs as protein scaffolds, RNA-binding protein (RBP) sponges, micro-RNA (miRNA) sponges, parental gene expression modifiers, alternative splicing regulators, and transcription regulators. To complement wet-lab experiments, considering the progress made by machine learning approaches for the determination of sub-cellular localization of other non-coding RNAs, the paper in hand develops a computational framework, Circ-LocNet, to precisely detect circRNA sub-cellular localization. Circ-LocNet performs comprehensive extrinsic evaluation of 7 residue frequency-based, residue order and frequency-based, and physio-chemical property-based sequence descriptors using the five most widely used machine learning classifiers. Further, it explores the performance impact of K-order sequence descriptor fusion where it ensembles similar as well dissimilar genres of statistical representation learning approaches to reap the combined benefits. Considering the diversity of statistical representation learning schemes, it assesses the performance of second-order, third-order, and going all the way up to seventh-order sequence descriptor fusion. A comprehensive empirical evaluation of Circ-LocNet over a newly developed benchmark dataset using different settings reveals that standalone residue frequency-based sequence descriptors and tree-based classifiers are more suitable to predict sub-cellular localization of circular RNAs. Further, K-order heterogeneous sequence descriptors fusion in combination with tree-based classifiers most accurately predict sub-cellular localization of circular RNAs. We anticipate this study will act as a rich baseline and push the development of robust computational methodologies for the accurate sub-cellular localization determination of novel circRNAs.

## 1. Introduction

**Figure 1:**
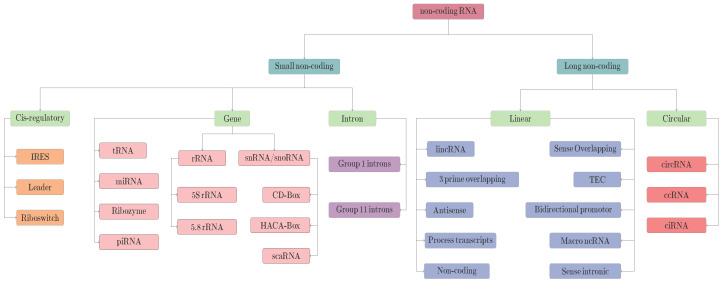
A Hierarchical Classification of Non-Coding RNAs.

Non-coding ribonucleic acids (ncRNAs) are now considered important regulatory elements instead of junk sequences [1]. Recent research findings have proved the significance of ncRNAs as they play important roles in diverse biological processes such as dosage compensation, cell differentiation, genomic imprinting [2], controlling gene expressions [3], and biomarker development [4]. ncRNAs gained more attention after the discovery of their association with diseases such as Alzheimer’s, cardiovascular diseases, and cancer [4]. To date, a number of different ncRNAs have been discovered with unique sequence nature, physical structures, and degree of contribution in diverse biological as well as physiological processes, a broad classification of which is provided in Figure 1. Circular RNA, a sub-type of long non-coding RNA, is emerging as a key player in the RNA world due to its crucial roles in multifarious cellular processes, aptitude to function as microRNA sponges, and regulation of gene transcription [5].

Although circRNAs in mammalian, plant viroids, and yeast cells were discovered around more than 50 years ago [6], they were often considered low abundance byproducts produced by RNA splicing [7,8]. These molecules were contemplated to be unlikely to perform any crucial role in important biological processes. Consequently, such molecules attained very little attention and only a handful of novel circRNAs were discovered until 2010 due to minimal research in biogenesis of circRNA [9]. However, with the advancement of biomedical research and development of RNA deep sequencing high-throughput technologies, more recently, several studies have changed the paradigm of belittling the biological significance of circRNAs [5,10,11,12].

Studies have proved that circRNAs can act as RNA-binding protein (RBP) sponges, micro-RNA (miRNA) sponges, parental gene expression modifiers, alternative splicing regulators, and transcription regulators, even a small number of circRNAs can be transformed into peptides or proteins as well [10,13,14,15]. Moreover, emerging evidence shows the involvement of circRNAs in cancer, atherosclerotic vascular ailment risk, and neurological disorders [16], and that they are abnormally expressed in colorectal cancer (CRC) [17]. circRNAs have been described as effective biomarkers for gastric cancer, aging, and as potential illness biomarkers in human saliva [18]. These findings indicate that circRNAs have immense potential to perform unique regulatory function in biological development, disease origination, and progression [13]. In addition, they are likely to become effective clinical diagnostic, symptomatic and prognostic markers to facilitate in-depth understanding related to the treatment of diverse convoluted diseases [13].

High-throughput sequencing analysis indicates that circRNAs reside in different sub-cellular compartments and primarily perform a variety of regulatory functions by co-localizing [19]. Like messenger RNA (mRNA), circRNAs are generated in nucleus and dominantly localized in cytoplasm [19]. Within the nucleus, circRNAs enhance the expression of mRNAs by binding to U1 snRNP and recruiting RNA polymerase II to promoter site of parental gene [10]. Moreover, nuclear circRNAs hampers pre-rRNA interaction with PES1 protein which alleviate the maturation and processing of rRNA [20]. circRNAs localize to cytoplasm perform different regulatory roles by interacting with proteins and miRNAS. Many circRNAs are found to act as miRNA sponges [21] and protein sponges [22] where circRNAs bind with corresponding miRNAs or proteins to minimize their inhibitory impact on their targets. A significant number of circRNAs are localized to the exosome which largely differ from other circRNAs present in the nucleus and cytoplasm of the respective cells [23]. Exosomal circRNAs act as targets for different diseases to facilitate effective diagnosis and treatment [24]. More recent studies have revealed the localization of circRNA in ribosome and their translation into endogenous peptides [25]. As sub-cellular localization of other ncRNAs has elucidated the role of ncRNAs in the functioning of neuronal dendrites [26], embryonic development [27], and gene regulation [28], as well as deepened our understanding of various scientific processes such as post-transcriptional regulation of genes [1], epigenetic functioning [1], protein–RNA interaction [1], and development and metabolism of cells [29]. Therefore, considering the multifarious biological roles of circular RNAs, accurate identification of circRNA sub-cellular localization patterns is essential to acquire deeper knowledge of molecular biology and the core functionality of diverse circRNAs.

Wet-lab experiments are still the most common approaches used to identify sub-cellular localization of different sub-types of ncRNAs. For instance, immunofluorescence confocal microscopy, immunoprecipitation, subcellular fractionation [30] are being used to determine sub-cellular localization of micro RNAs (miRNAs), affinity purification [31], ChIP-PCR [32] and Double-Luciferase Reporter assays [32] are being used for long ncRNAs (lncRNAs). Likewise, sub-cellular localization of circRNAs is determined through electron microscopy, RNA-sequence analysis, and quantitative polymerase chain reactions (qPCR) [33]. Where at one hand, these experimental approaches are expensive, resource-intensive, time consuming and less adaptable for a wide community of genomics researchers. On the other hand, researchers also need to employ additional validation technologies to assess the correctness of output [23]. These downfalls have made experimental approaches less appropriate for large-scale determination of circRNAs sub-cellular localization.

Emergence of diverse metathesauruses including RNALocate [34], Ensembl [35], and ENCODE [36] has opened new horizons for the large scale determination of sub-cellular localizations of different non-coding RNAs through computational methodologies [37,38]. To date, several long non-coding RNAs (lncRNAs) [39,40,41], miRNAs [42,43], and messenger RNAs (mRNAs) [42] sub-cellular localization prediction approaches have been presented. This progress has largely enhanced the elementary comprehension of molecular biology, the role of ncRNAs in crucial biological functions, their involvement in gene transcription, initiation and evolution of different diseases, and drug development [44,45]. For rising star circRNA, there exist a number of machine and deep learning-based approaches which can perform discrimination of circular RNAs from other non-coding RNAs, and circular RNA-protein binding site prediction [46]. However, sub-cellular localization of circRNA is under-studied [47] and according to our best knowledge, there does not exist any computational approach for circular RNA sub-cellular localization prediction.

Considering the downfalls of experimental approaches, there is a lack of computational circRNA sub-cellular localization approaches, and there is also the fact that sub-cellular localization prediction approaches developed for other ncRNAs [38] cannot be applied to determine the sub-cellular localization of circRNAs due to the differentiation of biological structure, distribution of residues, and sequence length. The paper in hand develops a computational framework (Circ-LocNet) capable of performing a large scale sub-cellular analysis of a variety of circRNAs. To accelerate the research concerning the development of computational approaches for circRNA sub-cellular localization prediction, it provides a benchmark performance for the task of circRNA sub-cellular localization by exploring the most widely used sequence descriptors and machine learning classifiers. Contributions of this work can be summarized as:

1. Development of a benchmark circRNA sub-cellular localization prediction dataset using public RNALocate database [34] which is comprised of 1,205 circRNA sequences annotated against 8 different sub-cellular localities.
2. A comprehensive performance analysis of residue frequency, residue order and frequency, and residue physicochemical property-based sequence descriptors is performed to find an appropriate standalone sequence descriptor for circular RNA sub-cellular localization prediction.
3. A detailed performance impact of K-order sequence descriptor fusion is performed by ensembling similar as well dissimilar genres of statistical representation learning approaches to reap the combined benefits in order to investigate whether sequence descriptor fusion significantly optimizes the statistical representation of circRNA sequences and which K-order sequence descriptor fusion manages to capture more discriminative residue distribution important for sub-cellular localization prediction.
4. Extensive empirical evaluation of 5 different generative, discriminative, and tree-based classifiers using various sequence descriptors is performed to investigate which type of classifier extracts a residue correlation that is important to accurately predict circular RNA sub-cellular localization.
5. An end-to-end computational framework (Circ-LocNet) that explores the performance of seven different standalone sequence descriptors, second-order sequence descriptor fusion, third-order sequence descriptor fusion, and so on all the way up to seventh-order sequence descriptor fusion with the five most widely used machine learning classifiers in order to determine an appropriate combination of sequence descriptor and machine learning classifier for circular RNA sub-cellular localization prediction.
6. Development and deployment of the very first interactive and user-friendly circRNAs sub-cellular localization prediction platform.

## 2. Results

This section illustrates the performance produced by proposed computational framework Circ-LocNet over the newly developed benchmark dataset using the seven most widely used sequence descriptors and five machine learning classifiers. Further, it describes the performance impact of K-order sequence descriptor fusion where it illustrates the best performance achieved by second-order, third-order, and going all the way up to seventh-order combination of sequence descriptor across five different machine learning classifiers.

### 2.1. Performance Assessment of Different Standalone Sequence Descriptors Using Distinct Genre Machine Learning Classifiers

Table 1 and Table 2 compare the top performance produced by seven different standalone residue frequency-based, residue order and frequency-based, and physico-chemical property-based sequence descriptors using the five most widely used machine learning classifiers for the task of circRNA sub-cellular localization prediction in terms of accuracy and F1-score, respectively. Performance analysis across both evaluation metrics reveals that, among all sequence descriptors, K-gap-based sequence descriptors such as TriMonoKgap and residue order and frequency-based sequence descriptors including PseudoKNC, K-mer achieved a top accuracy of 69% and F1-score of 63% followed by 68% accuracy, 62% F1-score, respectively, achieved by physico-chemical properties-based sequence descriptor EIIP, and simple folding curve-based sequence descriptor Z-curve showed the worst performance.

From all seven different sequence descriptors, five sequence descriptors (K-mer, RCKmer, PseudoKNC, EIIP, ZCurve,) achieve peak performance figures with Random Forest, whereas two sequence descriptors (TriMonoKgap, DiMonoKgap) achieve peak performance with XGBoost classifier, while TriMonoKgap, DiMonoKgap, EIIP, ZCurve, and PseudoKNC sequence descriptors produce the worst performance with Naive Bayes classifier; however, K-mer and RCKmer manage to produce decent performances with Naive Bayes classifier. Further, the majority of sequence descriptors produce a decent performance with Adaboost classifier, whereas no sequence descriptor manages to cross the performance of 50% with SVM classifier across both evaluation metrics.

**Figure 2:**
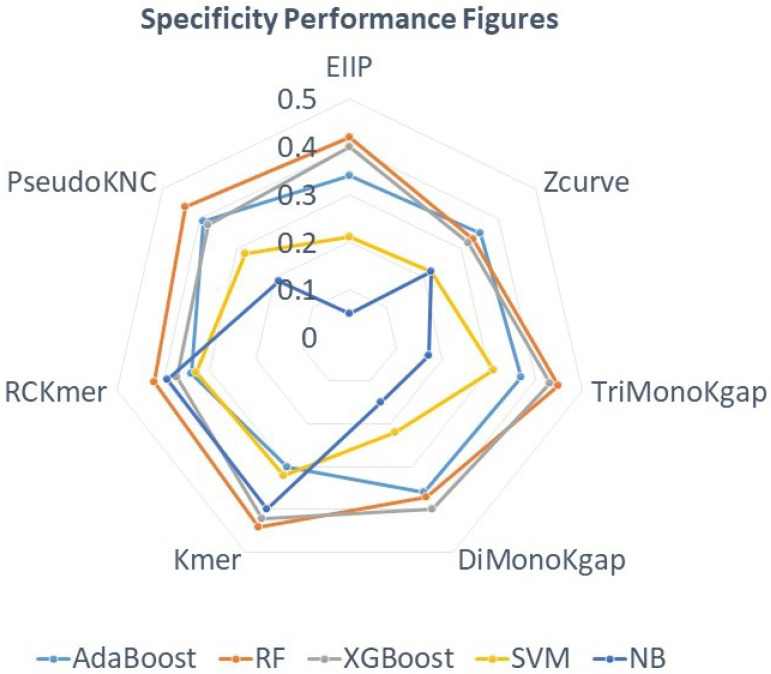
Standard Specificity Figures of 7 Different Sequence Descriptors Against 5 Different Classifiers.

In addition, to analyze the true negative rate of the seven different sequence descriptors with respect to five distinct machine learning classifiers, the performance trends of all sequence descriptors are mapped in a specificity spider chart (Figure 2).

Analysis of the polygon revealing specificity performance figures (Figure 2) shows that four sequence descriptors including PsuedoKNC, DiMonoKgap, TriMonoKgap, and EIIP mark better performance with decision tree-based machine learning classifiers (RF, AdaBoost, XGBoost), whereas three sequence descriptors such as ZCurve, K-mer, and RCKmer achieve higher specificity using discriminative (SVM) and generative (NB) machine learning classifiers. Among all machine learning classifiers, tree-based machine learning classifier RF achieves the highest specificity of 45% as well the second-best figure of 44% using two different sequence descriptors. From K-gap-based sequence descriptors, TriMonoKgap produces higher specificity across most machine learning classifiers as compared to DiMonoKgap. From K-mer-based descriptors, RCKmer marks better specificity with SVM and Naive Bayes, whereas PseudoKNC achieves better performance with AdaBoost and RF. However, in comparison to RCKmer and PsuedoKNC, K-mer achieves better specificity across most machine learning classifiers. On the other hand, simple sequence descriptors such as Z-curve and EIIP have shown quite similar specificity figures trends on AdaBoost and SVM, while Zcurve produces better performance than ZCurve across all machine learning classifiers except RF and XGBoost where EIIP manages to achieve better specificity. As a whole, ZCurve marks the peak of 35% using AdaBoost, which is better than its counterpart by 1%. From all sequence descriptors, TriMonoKGap marks quite consistent specificity across most classifiers followed by PseudoKNC, K-mer, and RCKmer. Overall, TriMonoKGap achieves the best performance mainly using tree-based machine learning classifiers, whereas EIIP shows tge worst performance mainly with SVM and NB classifier.

**Figure 3:**
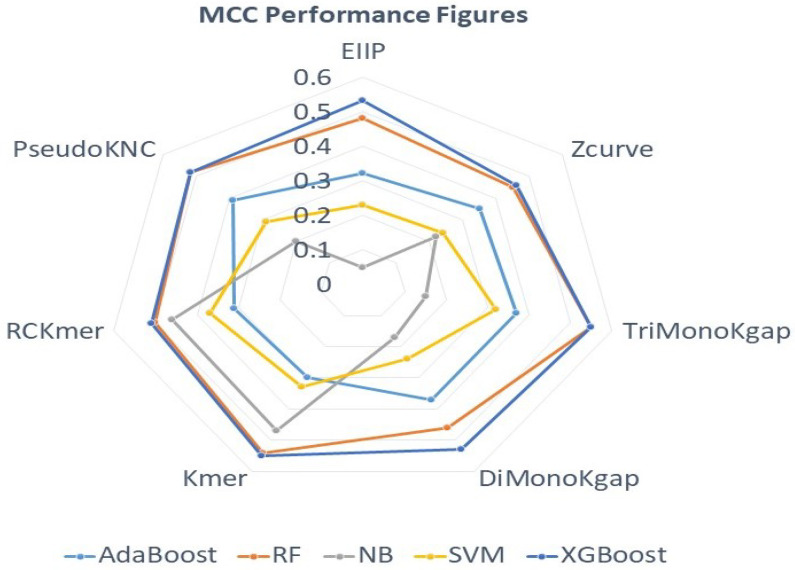
Standard MCC Figures of 7 Different Sequence Descriptors Against 5 Different Classifiers.

In order to assess the overall effectiveness of seven sequence descriptors across five different machine learning classifiers mainly by taking all four true positives, true negatives, false positives, and false negatives into account, the performance trends of all sequence descriptors are mapped to MCC (Figure 3) spider charts.

Assessment of the spider chart (Figure 3) revealing MCC figures of distinct sequence descriptors across five different machine learning classifiers reveals that all sequence descriptors achieve the highest MCC with decision tree-based machine learning classifiers. Among all tree-based machine learning classifiers, XGBoost achieves the highest performance followed byRF and AdaBoost, whereas discriminative machine learning classifier SVM and generative machine learning classifier NB mark a lower performance. Analyzing the MCC figures with respect to sequence descriptors, from Kgap-based approaches, once again TriMonoKgap not only produces better performance across most machine learning classifiers than DiMonoKgap but also outperforms the peak MCC performance of DiMonoKgap by 2% through achieving the peak of 55%. All K-mer-based sequence descriptors attain a better performance with XGBoost and RF. However, Kmer attains the peak MCC of 55% followed by 52% of PseudoKNC, and 51% of RCKmer using XGBoost classifier. Simple sequence descriptors including Z-curve and EIIP mark lower performance across most machine learning classifiers. However, EIIP manages to attain a peak MCC figure of 53% with XGBoost classifier which is better than the Z-curve peak performance by 7%. Among all sequence descriptors, TriMonoKgap and Kmer achieve the highest MCC figure of 55% using XGBoost classifier.

**Figure 4:**
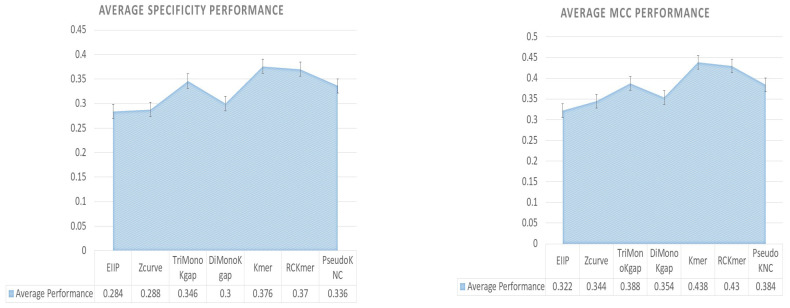
Average Specificity and MCC Figures of 7 Different Sequence Sequence Descriptors Against 5 Different Classifiers.

Further, to demonstrate the overall effectiveness of each sequence descriptor by aggregating their performance impact across all five machine learning classifiers, average specificity (Figure 4) and MCC (Figure 4) performance figures are shown in area charts. As is indicated by Figure 4, on average, Kmer achieves the highest specificity of 38% followed by 37% by RCKmer, and 34% by TriMonoKgap and PseudoKNC. DiMonoKgap marks an average specificity of 30% which is 1% better than ZCurver performance. Among all sequence descriptors, EIIP achieves the lowest average specificity figures. Similarly, analysis of average MCC figures (Figure 4) shows that, on average, Kmer and RCKmer achieve the highest performance of 43%, followed by 38% of TriMonoKgap and PseudoKNC. DiMonoKgap marks the average MCC of 35% which is slightly better than ZCurve. Among all, once again EIIP produces the lowesr average MCC figures.

**Figure 5:**
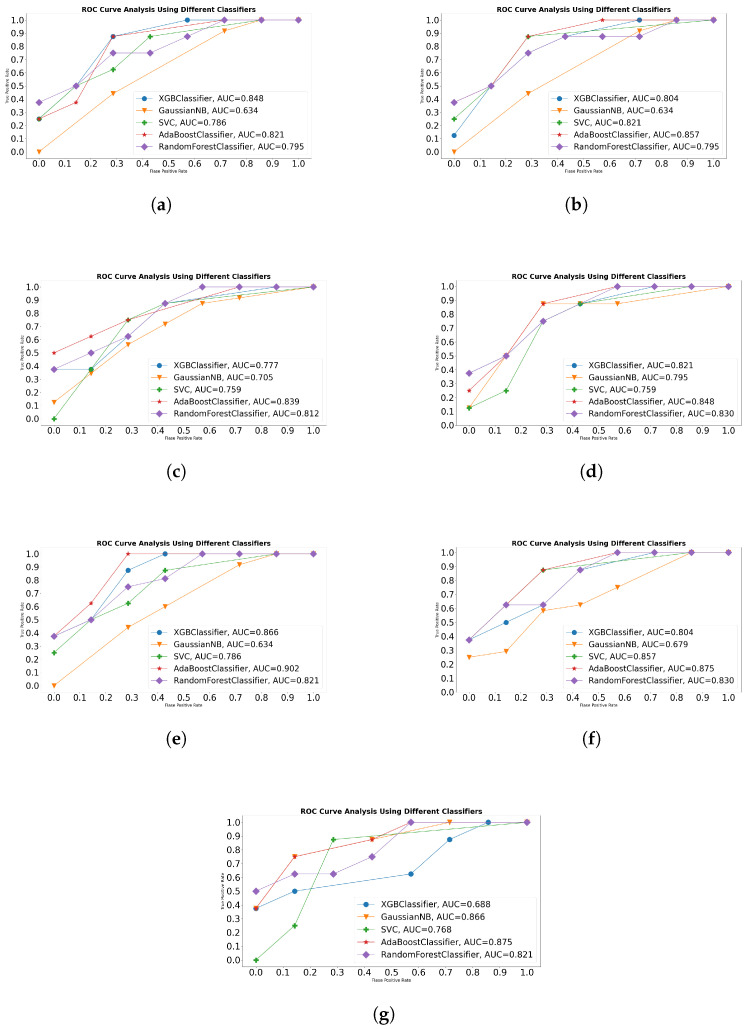
AU-ROC Performance Figures Produced by 5 Different Classifiers Using 3 K-gap, 3 K-mer and 2 simple Sequence Sequence Descriptors on a Benchmark Circular RNA Sub-Cellular Localization Dataset, (**a**) TriMonoKgap Peak Performance using 5-mers, (**b**) DiMonoKgap Peak Performance using 2-mers (**c**) RCKmer Peak Performance using 5-mers, (**d**) Kmer Peak Performance using 5-mers, (**e**) PseudoKNC Peak Performance using 5-mers, (**f**) EIIP Peak Performance, (**g**) Z-Curve Peak Performance.

In addition, considering the unique functional paradigm of seven different sequence descriptors, we analyze the impact of different sequence descriptors over the generalizeability of a variety of machine learning classifiers using area under receiver operating characteristic (AU-ROC). Using One-Versus-All setting, receiver operating characteristics (ROC) curves along with peak AU-ROC scores produced by frequency based and physico-chemical property-based sequence descriptor with optimal parameters against five machine learning classifiers are shown in Figure 5.

Critical analysis of ROC curves indicates that both K-gap based sequence descriptors show promising performance with decision tree-based classifiers including AdaBoost, XGBoost, Random Forest and discriminative classifier SVM, whereas a combination of K-gap based sequence encoding schemes with generative classifier NB does not prove particularly fruitful. Among all, DiMonoKgap in combination with AdaBoost achieves slightly better degree of separability (86%) as compared to 85% degree of separability achieved by TriMonoKgap using XGBoost classifier. Further, most machine learning classifiers manage to attain a decent AU-ROC score of 80% using K-gap based sequence descriptor. Similarly, all 3 K-mer based sequence descriptors also achieve a better degree of separability with decision tree-based classifiers and discriminative classifiers as well as producing the lowest AU-ROC scores with generative classifier NB. RCKmer manages to cross the mark of 80% using two classifiers, Random Forest and AdaBoost, and K-mer performs even better by achieving higher degree of separability across most machine learning classifiers and crossing the figure of 80% using XGBoost, Random Forest, and AdaBoost classifiers. K-mer attains a peak AU-ROC score of 85% which supersedes RCKmer peak score by 1%. Among all K-mer-based sequence descriptors, PseudoKNC achieves highest degree of separability across most machine learning classifiers, attaining a peak AU-ROC score of 90% with AdaBoost classifier and 86% with XGBoost classifier. Comparing fold curve and physico-chemical property-based sequence descriptors across all five machine learning classifiers reveals that EIIP attains better AU-ROC scores across most classifiers as compared to Z-Curve. Just like other sequence descriptors, EIIP manages to produce promising performance with decision tree-based and discriminative classifiers, achieving the peak AU-ROC of 88% using AdaBoost classifier, whereas Z-Curve crossed the figure of 80% using two decision-tree-based and one generative classifier (NB). Z-Curve attains the peak AU-ROC score of 88% using AdaBoost and the only sequence descriptor which produces a promising performance using NB classifier. Z-Curve in combination with NB produces an AU-ROC score of 87% which outperforms other sequence descriptor-based generative classifier performances by 7%. Among all combinations of sequence descriptors and machine learning classifiers, PseudoKNC achieves the highest AU-ROC score using AdaBoost classifier followed by 88% achieved by Z-Curve and EIIP.

To summarize, TriMonoKgap marks the best performance with Random Forest and XGBoost classifiers across most evaluation metrics, whereas Kmer and RCKmer produce better aggregated performance computed using five different classifiers.

### 2.2. Performance Assessment of K-Order Sequence Descriptors Fusion Using Distinct Genre Machine Learning Classifiers

Statistical representation learning paradigms of residue frequency-based, residue order and frequency-based, and physico-chemical properties-based sequence descriptors largely differ from one another in terms of coverage of residue contextual information, ability to capture residue semantic relatedness, short and long range dependencies, extraction of discriminative features, and residue correlation distribution important to predict sub-cellular localization of circRNAs. We were inspired by the fact that ensemble learning effectively handles bias-variance trade-off to largely improve the robustness and performance of predictive modelling by strategically combining multiple models. In order to generate optimized representation of circRNA sequences, instead of solely relying on standalone sequence descriptors, we perform comprehensive experimentation with K-order sequence descriptor fusion where we integrate similar as well dissimilar genre of sequence descriptors to reap the combined benefits. Mainly, we generate second-order, third-order, and go all the way up to seventh-order combinations of sequence descriptors to comprehensively investigate whether sequence descriptor fusion manages to optimize representation of circRNAs as well as which K-order sequence descriptor fusion most effectively captures the characteristics of sequence residues important to improve circRNA sub-cellular localization prediction performance.

To perform K-order sequence descriptor fusion, using seven different sequence descriptors, all possible K-order combination of sequence descriptors are generated and fed to five different machine learning classifiers. For instance, for second-order sequence descriptor fusion, from seven sequence descriptors, every sequence descriptor is combined with only one other sequence descriptor, whereas in third-order sequence descriptor fusion, every sequence descriptor is combined with two other sequence descriptors, and so on. As we have performed experimentation with seven different sequence descriptors, the value of K ranges from two tp seven.

To better illustrate the results, Table 3 indicates the best-performing K-order sequence descriptor fusion across five different machine learning classifiers. Performance figures of only the best-performing K-order sequence descriptor fusion across five distinct machine learning classifiers are provided in terms of accuracy and F1-score in Table 4 and Table 5, respectively. An in-depth performance analysis indicates that for tree-based classifier Random Forest, among many second-order sequence descriptor fusion approaches, residue frequency-based TriMonoKGap in combination with residue order and frequency-based PseudoKNC mark the best performance, achieving an accuracy of 70% and F1-score of 64%. For Random Forest classifier, the performance of other K-order sequence descriptor fusions also produce similar figures, indicating that Random Forest achieves peak performance with second-order sequence descriptor fusion. Another tree-based classifier, XGBoost, also achieves its peak accuracy of 69% and an F1-score of 64% on second-order (Kmer+diMonoKGap) and third-order sequence descriptor fusion (pseudoKNC+diMonoKGap+RCKmer), where the performance of other K-order sequence descriptor fusions fall very close to peak figures. Among all tree-based machine learning classifiers, AdaBoost achieves the lowest performance in terms of accuracy and F1-score across six different K-order sequence descriptor fusions. It achieves the top accuracy and F1-score of 58% with third-order sequence descriptor fusion where the combination of diMonoKGap, pseudoKNC and zCurve proves fruitful. Similarly SVM classifier achieves top accuracy of 68% and F1-score of 62% third-order sequence descriptor fusion where the integration of diMonoKGap, EIIP, and zCurve sequence descriptor outperforms other combinations. Naive Bayer classifier attains the highest accuracy of 61% and an F1-score of 59% with second-order sequence descriptor fusion where the combined benefits of RCKmer and Kmer are reaped. However, unlike other machine learning classifiers, Naive Bayes is the only classifier where the performance of other K-order sequence descriptor fusions plunge below 25%. Using six different K-order sequence descriptor fusions, comparing the performance of machine learning classifier reveals that overall Random Forest achieves a better performance followed by XGBoost and SVM classifier. Among all tree-based classifiers, AdaBoost marks the lowest performance; however, it still manages to achieve decent performance across most K-order sequence descriptor fusions, whereas Naive Bayes achieves better peak performance than AdaBoost, but its performance significantly drops in most K-order sequence descriptor fusions.

In a nutshell, the idea of K-order sequence descriptor fusion proves fruitful as Random Forest classifier manages to outperform the accuracy of 69% and F1-score of 63% achieved through standalone sequence descriptors by 1% through fusing TriMonoKgap and PseudoKNC sequence descriptors, while tree-based machine learning classifiers (Random Forest, XGBoost, AdaBoost) and SVM classifier achieve peak performance with the combination of heterogeneous sequence descriptors. Naive Bayes classifier achieves peak performance with the combination of quite similar sequence descriptors. Overall, most machine learning classifiers mark better performance with second-order and third-order sequence descriptor fusion. More specifically, Random Forest and Naive Bayes classifier achieve better performance with second-order sequence descriptor fusion, whereas XGBoost, AdaBoost, and SVM classifier achieve better performance with third-order sequence descriptor fusion.

## 3. Discussion

Considering the fact that circRNAs perform a myriad of regulatory functions mainly by co-localizing, accurate determination of circRNA sub-cellular localization is indispensable to understand their association with diverse physiological and pathological processes as well as their potential for biomarker development. A significant number of computational approaches have been developed for sub-cellular localization of different ncRNAs (e.g., LncRNA, miRNA) which primarily leverage a unique combination of a sequence descriptor and machine learning classifier. Evidently, raw sequence-based computational approaches are more scalable, efficient, and appropriate for large-scale determination of ncRNA sub-cellular localization as they do not rely on any expensive resources (e.g., expression profile). However, no researcher has assessed whether residue distribution of circRNA sequences is also informative enough for the determination of circRNA sub-cellular localization. Further, which genre of sequence descriptors and machine learning classifier are more suitable to capture residue semantic relatedness, short- and long-range residue dependencies, positional invariance of residues, and other residue characteristics important for circRNAs sub-cellular localization prediction has not been determined.

In this regard, the proposed Circ-LocNet framework explores the performance potential of the seven most extensively used sequence descriptors using five different machine learning classifiers under the hood of two different settings. In the setting of using a standalone sequence descriptor in combination with a machine learning classifier, residue frequency-based sequence descriptors ((TriMonoKgap, Kmer, RCKmer, PseudoKNC)) most effectively characterize circRNA sequences and tree-based machine learning classifiers best exploit the residue hidden correlations to predict sub-cellular localization of circRNAs. Across all K-gap and K-mer based sequence descriptors, performance is improved with the increase of K-mers or K-gap values. Most residue frequency-based sequence descriptors achieve higher performance with five mers or five gaps. In other settings, the predictive performance of tree-based machine learning classifiers is further improved by K-order sequence descriptor fusion. It is seen that second-order or third-order fusion of heterogeneous sequence descriptors generates more discriminative statistical representation circRNA sequences.

Furthermore, considering the small size of the benchmark circRNA dataset and the fact that deep learning classifiers perform better with large training data, in this study, we have performed experimentation with only traditional machine learning classifiers. Besides this, the reasons for not comparing the performance of circular RNA subcellular localization prediction model with subcellular localization prediction models developed for other non-coding RNAs are manifold. First, unlike most non-coding RNA sequences, circRNA sequences are not linear by nature. Moreover, circRNA sequences differ from other non-coding RNA sequences in terms of sequence lengths, distribution of nucleotides, etc. [48]. Besides this, most non-coding RNA subcellular localization determination tasks (e.g., miRNA/lncRNA/snoRNA subcellular localization) and predictors are of the multi-label type, whereas the circRNA subcellular localization determination task and predictor are of the multi-class type [48]. Due to these differences, we have not compared the performance of circular RNA subcellular localization predictor with existing non-coding RNAs subcellular localization predictors.

In a nutshell, we consider this a pioneering work of benchmarking the performance of different genre sequence descriptors and machine learning classifiers that will open new horizons for the computational determination of circRNA sub-cellular localization.

### Interactive and User-Friendly Circ-Locnet Web Server

In order to facilitate biomedical researchers and practitioners, we have developed the very first user-friendly web sever for the proposed circRNA sub-cellular localization prediction framework (Circ-LocNet). This web server is available at (https://circ_rna_location_predictor.opendfki.de/) and can be used to find sub-cellular localities of circRNAs of different species as well as to validate experimentally identified sub-cellular localities by only using raw circRNA sequences. Unlike web servers developed to categorize biomolecules, find sub-cellular locations, and to predict intera-specie or inter-specie interaction, this web server allows the users to train and optimize different machine learning classifiers from scratch, and perform inference on novel circRNA sequences belonging to existing or new specie under different settings.

## 4. Materials and Methods

This section describes the workflow of proposed machine learning based Circ-LocNet framework. It also sheds light on the collection and preparation of benchmark dataset and evaluation metrics used to assess the performance of Circ-LocNet framework.

### 4.1. Circ-Locnet

**Figure 6:**
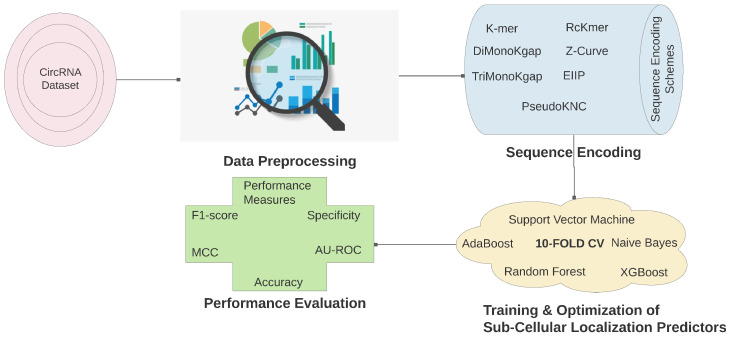
CircLoc-Net: A Computational Framework for Sub-cellular Localization Prediction of circRNAs.

Proposed computational framework Circ-LocNet explores different sequence descriptors and machine learning classifiers to develop an optimal pipeline for circular RNA sub-cellular localization, workflow of which is demonstrated in Figure 6. Selection criteria of sequence encoding approaches, their working paradigm, and functioning principles of diverse sub-cellular localization predictors used by Circ-LocNet are discussed in detail in the following sub-sections.

#### 4.1.1. Sequence Encoding Approaches

The prime goal of sequence encoding approaches is to formulate the genomic or proteomic sequences with rich mathematical expression which can effectively reflect their crucial correlation with the concerned target [49]. Given a genomic or proteomic sequence S, its straightforward expression can be written as:

(1) $$S=R_{1},R_{2},R_{3},R_{4},R_{5},R_{6},R_{7},R_{8},.......,R_{L}$$

where R represents the residue and subscript denotes the position of residue in the sequence of length L. Since all of the most widely used machine learning algorithms including Support Vector Machine, K-Nearest Neighbor, Naive Bayes, Principal Component Analysis, Random Forest operate on fixed-length sequence vectors [49], we have to transform sequential expression given in Equation (1) into real-valued vectors. Considering the unique nature of genomic and proteomic sequences as well as distribution of residues in sequences, a variety of sequence descriptors are being used to encode the biological characteristics of residues into rich statistical vectors [49]. Statistical representation learning of raw sequences is an important sub-task of sequence analysis pipeline. A better descriptor that generates the statistical vector by taking both residue frequency and semantic information into account can largely assist the classifier in discriminating between sequences of diverse classes. On the other hand, a too-simple statistical representation learning descriptor can largely deteriorate the generalizeability of classifier by generating very similar representation for sequences of different classes. As sequence descriptors mainly rely on nature and different characteristics of sequences, as well as the distribution of residues within sequences [49], their ability to learn effective representation across different genres of sequences largely fluctuates. For instance, a sequence descriptor which effectively encodes the important information for miRNA [43] or piRNA sequences [50] might fail to capture important information in lncRNA or circRNA sequences. This is because miRNA [43] and piRNA sequences are shorter in length [50], having residues in the range of 17 to 25, whereas lncRNA [39,40] or circRNA sequences are larger in length, having residues in the range of 213 to 22,743 [51]. For sequences of longer lengths, we need sequence descriptors that can effectively capture long range dependencies of residues. Here, for the task of circRNA sub-cellular localization, we introduce 7 different sequence descriptors which are extensively being used to encode ncRNA sequences that are characteristically similar to circRNA sequences [52]. Under different settings, we practically explore the effectiveness of different descriptors for learning better statistical representation of residues and enhancing the ability of 5 distinct machine learning classifiers for the circRNA sub-cellular localization prediction task. Different sequence residue descriptors used in this study are briefly described in following sub-sections.

#### 4.1.2. K-Mer

**Figure 7:**
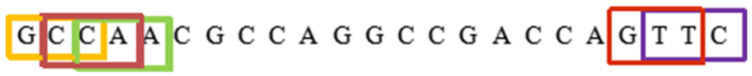
Process of Generating Sequence K-mers (e.g., 3-mers), Where each Particular Color Frame Denotes a Unique 3-mer.

Among many sequence encoding approaches, K-mer is the most common and simplistic sequence encoding approach to represent DNA, RNA, or protein sequences [53]. Figure 7 illustrates the process of generating k-mers using a window size of 3 and stride of 1. K-mer is applied for diverse genomic sequence analysis tasks including enhancer identification [54], human gene regulatory sequence prediction [55,56], and regulatory sequence features determination [57]. Likewise, we can compute k-mers frequency information in circRNA sequences. For any sequence having length m, number of k-mer sub-sequences of length k can be computed by m−k+1. Usually, circRNA sequences consist of 4 bases, A, U, C, G; hence, k-mers of length 4 have 4K total structures. For each circRNA sequence, it generates a feature vector by taking the frequencies of k-neighboring residues into account [53].

#### 4.1.3. Reverse Compliment Kmer

Reverse Compliment Kmer is a variant of simple k-mer residue encoding approach which is utilized by several researchers to predict regulator region of DNA (Promoters) [58], DNA N4-methylcytosine sites [59], and RNA-associated sub-cellular localizations [60]. It first reverses the order of residues present in RNA sequences and then computes the compliment of every residue to generate new RNA sequences [55]. The encoding of each residue is computed by normalizing the count of Kmers present in updated sequences [55]. For instance, for the basic 4 residues, AGCU, considering the *k* = 2, 16 k-mers can be generated ‘AA’, ‘AC’, ‘AG’, ‘AU’, ‘CA’, ‘CC’, ‘CG’, ‘CU’, ‘GA’, ‘GC’, ‘GG’, ‘GU’, ‘UA’, ‘UC’, ‘UG’, ‘UU’; however, by eliminating the reverse complementary k-mers, only 10 unique k-mers are available in reverse complementary k-mer encoding approach ‘AA’, ‘AC’, ‘AG’, ‘AU’, ‘CA’, ‘CC’, ‘CG’, ‘GA’, ‘GC’, ‘UA’. In our experimentation, 2 to 5-mers are generated to better illustrate the impact of different k-mers in capturing inherent residue relationships present in circRNA sub-cellular localization sequences.

#### 4.1.4. Psuedoknc

Genomic sequences are comprised of 4 basic residues (A, C, G, T/U). For a genomic sequence of merely 100 residues, number of possible residue order combinations are $4^{100log4}$ > 16,065 ∗ $10^{60}$. However, considering that genomic sequences are far greater than 100 residues, possible residue order combinations are significantly greater than 16,065 ∗ $10^{60}$ value. It seems impracticable to statistically cover all possible diverse residue order patterns. Moreover, genomic sequences significantly vary in length, which makes the incorporation of residue order information even more difficult. To effectively handle such phenomena, researchers have been looking for a residue encoding scheme capable of partially incorporating the residue order effects. Building on the fact that proteomics sequence residues also face a similar problem, Chou et al. [61] developed pseudo amino acid composition (PseAAC) for proteomics sequences. Following the wide success of PseAAC in diverse proteomics sequence analysis tasks [62,63,64,65], researchers have developed Psuedo K-tuple based nucleotide composition (PsuedoKNC) to effectively incorporate contiguous local and global sequence order information of k-tuples of genomic sequences [66,67,68]. PseudoKNC is utilized by several researchers to predict DNA N6-methyladenosine sites [69], nucleosome posistioning in genomes [66], and promoters in prokaryotes [70].

Given an RNA sequence D having L nucleotides:

(2) $$\begin{array}{c} D=N_{1},N_{2},N_{3},N_{4},......N_{L},\;where\;N_{i}\in adenine(A),guanine(G),cytosine(C),Uracil(U) \end{array}$$

here, $N_{i}$ represents nucleotide at specific position (i = 1, 2, …, L) in the sequence D. In this case, if the RNA sequence is represented by Di-Nucleotide composition, we obtain:

(3) $$D={\left[f(AA)f(AC)f(AG)f(AU)......f(UU)\right]}^{T}={\left[f_{1}^{di}f_{2}^{di}f_{3}dif_{4}^{di}........f_{16}^{di}\right]}^{T}$$

where the letter T represents transpose operator, $f_{1}^{di}=f\left(AA\right)$ refers to the normalized occurrence frequency of AA in RNA sequence, $f_{2}^{di}=f\left(AC\right)$ is the normalized occurrence frequency of AC in RNA sequence, and so forth. Similarly if RNA sequence is represented with tri-nucleotide composition, we get:

(4) $$D={\left[f(AAA)f(AAC)f(AAG)f(AAU)......f(UUU)\right]}^{T}={\left[f_{1}^{tri}f_{2}^{tri}f_{3}trif_{4}^{tri}........f_{64}^{tri}\right]}^{T}$$

where $f_{1}^{tri}=f\left(AAA\right)$ refers to the normalized occurrence frequency of AAA in RNA sequence, $f_{2}^{tri}=f\left(AAC\right)$ is the normalized occurrence frequency of AAC in RNA sequence, and so forth. Generically, if an RNA sequence is represented through K-tuple nucleotide composition, sequence vector D for RNA sequence will have $4^{K}$ components such as:

(5) $$D={\left[f_{1}^{K-tuple}f_{2}^{K-tuple}f_{3}^{K-tuple}....f_{4^{K}}^{K-tuple}\right]}^{T}$$

In this study, we experiment with di-nucleotide, tri-nucleotide, tetra-nucleotide, and penta-nucleotide compositions to represent circRNA sequences for the task of circRNA sub-cellular localization prediction.

(6) $$\left[\begin{array}{cc} PseKNC=Pseudodinucleotidecomposition(PseDNC) & When\;K=2\\ Pseudotrinucleotidecomposition(PseTNC) & When\;K=3\\ Pseudotetranucleotidecomposition(PseTTNC) & When\;K=4\\ Pseudopentanucleotidecomposition(PsePNC) & When\;K=5 \end{array}\right]$$

#### 4.1.5. Z-Curve

The Z-curve residue encoding approach was proposed by Zhang et al. [71] in which DNA or RNA sequences are mapped into the folding curve using 3-dimensional space. The Z-curve encoding approach was developed using the symmetry of traditional tetra-hedrons [71]. It is being extensively applied for gene identification and DNA/RNA sequence analysis [72], recognition of prokaryotic promoters [73], replication origins in archaeal genomes [74], identification of protein coding genes within bacterial and archaeal genomes [75]. Z-curve comprises of 3 components (x, y, z) where each component has particular biological significance. More specifically, x component represents the dispersion of purine (A + G) to pyrimidine (C + T) across the DNA/RNA sequences. Higher proportion of purine bases than pyrimidine base indicates x > 0, lower percentage is shown using x < 0, and equal distribution is represented as x = 0. Likewise, y indicates the dispersion of amino pair (A + C) with respect to ketone (G + T) across DNA/RNA sequences. Among all components, z represents the distribution of feeble hydrogen bonds (A + T) to powerful hydrogen bonds (G + C) across DNA/RNA sequences. These three Z-curve components have the distribution information of residues present in DNA/RNA sequences [76]. Mathematically, zCurve computation can be represented as follows:

(7) $$\begin{array}{c} x=(\sum (A)+\sum (G))-(\sum (C)+\sum (U))\\ y=\left(\sum \left(A\right)+\sum \left(C\right)\right)-\left(\sum \left(G\right)+\sum \left(U\right)\right)\\ z=\left(\sum \left(A\right)+\sum \left(U\right)\right)-\left(\sum \left(G\right)+\sum \left(C\right)\right) \end{array}$$

#### 4.1.6. Electron–Ion Interaction Pseudopotentials of Trinucleotide (Eiip)

EIIP is a physico-chemical property-based encoding scheme that represents the dissemination of electronion energies across the sequence. EIIP was originally given by Nair et al. [77] to convert DNA sequences into vector space. Unlike k-mer frequency based residue encoding schemes, EIIP is a lightweight yet very powerful approach which does not require any hyperparameter tuning to better capture any residue order and local–global context [78,79]. While generating sequence representation, EIIP uses pre-computed float value for each distinct residue. State-of-the-art residue representation generation toolkit iLearnPlus [49] considers EIIP values (A, 0.1260; C, 0.1340; G, 0.0806; and T, 0.1335) to encode only DNA sequences. However, more recently, Dou et al. [80] have explored the suitability of 8 diverse residue encoding schemes including EIIP to obtain inherent dependencies of RNA sequence residues for m5c modification prediction task [80]. To generate the statistical representation of RNA sequence residues, they considered Thymine (T) equivalent to Uracil (U) to leverage EIIP values (A = 0.1260; C = 0.1340; G = 0.0806; and U = 0.1335). Considering the extensive adoption of EIIP for biomedical sequences classification [81,82], gene prediction [83], cancer classification [84], exons location prediction [85], and promoters prediction [86], here we utilize EIIP to transform circRNA sequences into vector space. Mathematically, the paradigm of EIIP for encoding circRNA sequences can be represented as follows:

(8) $$V_{EIIP}=\left[EIIP_{A}⊕EIIP_{C}⊕EIIP_{G}⊕EIIP_{U}\right]$$

#### 4.1.7. Xxkgap

XxKGAP composition makes use of kgaps present in residue sub-sequences. Occurrences of sub-sequences are taken as prediction features [49]. Researchers have utilized Kgap based encoding schemes to infer m5C Modifications in RNA Sequences [80], enhancers [87], pre-miRNA [88], and to analyze medical records [89]. In this study, we experiment with DiMonoKgap, and TriMonoKGap with kgap ranging from 2-to-5. Mathematically, working of DiMonoKgap, and TriMonoKGap can be expressed as follows:

(9) $$C_{dmkgap}=WX\_kgap\_Y\;,\;C_{tmkgap}=WXY\_kgap\_Z\;kgap>0$$

(10) $$Sequence_{vec}=\Theta_{unq=1}^{n}|WX\_Y_{unq}|\subset C_{d}mkgap\;,\;\Theta_{unq=1}^{n}\left|WXY\_Z_{unq}\right|\subset C_{t}mkgap$$

Here (in Equation (9)), $C_{dmkgap}$ represents collection of DiMonoKgap residues and $C_{tmkgap}$ refers to collection of TriMonoKGap residues where second-order (WX) and third-order residues (WXY) combined with first-order residues (Y, or Z) taken using positive kgap value. As is indicated by Equation (10), for each residue encoding scheme (DiMonoKgap, TriMonoKGap), sequence vectors ($Sequence_{vec}$) are generated by concatenating the count of unique second-order–first-order residues ($WX\_Y_{unq}$) or third-order–first-order residue ($WXY\_Z_{unq}$) using respective collections ($C_{dmkgap}$, $C_{tmkgap}$).

#### 4.1.8. Circular RNA Sub-Cellular Localization Predictors

In order to better illustrate the effectiveness of diverse sequence descriptors and to facilitate a rich baseline for circRNA sub-cellular localization task, we perform experimentation with 5 most widely used machine learning classifiers. Specifically, we employ Random Forest (RF), Support Vector Machine (SVM), AdaBoost, XGboost, and Naive Bayes (NB) classifiers, a brief description of which is given below.

We utilize an efficient discriminative classifier known as SVM [90] for circRNA sub-cellular localization prediction. SVM has been extensively utilized for regression, outlier detection, and classification in diverse fields including Natural Language Processing (NLP) [91], Genomics, Proteomics, and Bioinformatics [92]. SVM performs classification by projecting independent variables into high-dimensional feature space such that classes are linearly separable [90]. Because SVM is categorized as a binary classifier, therefore, one-against-one or one-against-all approach is typically used for multi-class classification problems. In one-against-one approach, k(k-1)/2 binary classifiers are trained where each classifier learns to discriminate a distinct pair of k classes. For inference, the class with majority vote is chosen as the final prediction. However, in the one-against-all paradigm, the multi-class classification problem is transformed into a binary classification problem where the actual class label is treated as positive and all other class labels are treated as negative to train K binary classifiers. For inference, among all binary classification models, the model with the highest confidence is used. In order to transform feature vectors into superior Hilbert space, SVM makes use of kernel trick. Using one-against-all strategy, we have experimented with Polynomial Kernel, Radial Basis Function (RBF), and Gaussian Kernel, however we find that SVM with RBF kernel finds best the hyperplane for circRNA sub-cellular localization prediction [92].

Naive Bayes [93] is a simple supervised machine learning algorithm which computes class probabilities using Bayes theorem while assuming that all corpus features are fully independent. During inference, Naive Bayes predicts those classes which have highest probabilities. To compute probabilities from the collection of continuous features, it is indispensable to estimate their probability distributions which is usually done by kernel density estimation [93]. Although the assumption of considering all features independent by Naive Bayes rarely holds in practice; however, Naive Bayes has shown competitive performance in comparison to more advanced classifiers for diverse NLP [94] and Bioinformatics tasks [95]. Considering the dominant utilization of Gaussian kernel density, in our experimentation, we implement Gaussian Naive Bayes method for circRNA sub-cellular localization prediction task.

Classification trees are also used to predict sub-cellular localization of circRNAs using sequence information. Classification trees leverage a tree-like data structure for efficient sequence classification. Nodes of the tree denote binary decision rules that recursively segregate the feature space whereas the leaves of the tree denote the classes [96]. Classification trees are highly interpretable and really effective in dealing nonlinear relationships as well as interactions among the variables. However, they are very sensitive to noisy data and are more vulnerable to overfitting [97]. Tree-based ensemble learning approaches usually combine several classification trees to formulate highly stable and more accurate classification pipelines than standalone classification trees [98]. Boosting and Bagging are the 2 most widely used applications of tree-based ensemble learning. In boosting, sequence of classification trees are trained where each successive classification tree aims to reduce training errors by fixing the wrong classifications of preceding trees. Inference is accomplished through weighted voting between all classification trees, whereas in bagging, several classification trees are trained in a parallel manner using bootstrap samples of sequences. For inference, the final class is estimated by a majority between all trees. In our experimentation, we utilize 3 tree-based ensemble approaches, among which are 2 are based on the boosting paradigm and 1 is based on the bagging paradigm.

We utilize a renowned tree-based bagging ensemble called Random forest [99], where the overall predictive performance is improved by effectively combining several decision tree predictors. It has been employed by several researchers to achieve promising performance for diverse Natural Language Processing [94] and bioinformatics tasks [92].

The first boosting ensemble applied in this study, “AdaBoost", fits a number of weak learners (short decision trees which are slightly superior than random guessing models) on iteratively modified version of underlay data. Afterwards, predictions made by all classifiers on different versions of the dataset are combined using weighted majority voting paradigm to generate the final prediction.

Considering the smart penalisation of trees, overall network boosting, proportional compression of leaf nodes, and an effective parameter randomization of renowned XGBoost classifier, we also adapt XGBoost for sub-cellular localization prediction of circRNAs. Several researchers have shown the effectiveness of XGBoost classifier for diverse NLP and bioinformatics tasks [92].

### 4.2. Circular RNA Sub-Cellular Localization Dataset

**Figure 8:**
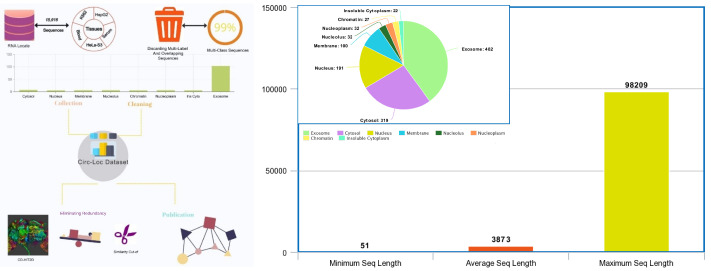
Workflow of Generating circular RNA Sub-cellular Localization dataset comprised of following steps: Collecting raw sequences and associated sub-cellular localization’s, Eliminating Redundancy, and Transforming the dataset into Standard format. Bar Chart and Pie Graph illustrates Statistics of Dataset.

The generation of a benchmark corpus is indispensable to develop machine learning-based applications [100]. We have utilized RNALocate database [34] to develop a circular RNA sub-cellular localization dataset. In the RNALocate database [34], we have found 59,161 circular RNA symbols. Using these symbols, we have found 15,916 sequences of 5 different tissues (K562, HepG2, Blood, HeLa-S3, Serum) that belong to 8 different sub-cellular locations. A comprehensive analysis of extracted sequences and associated sub-cellular locations revealed that 109 sequences belong to more than one sub-cellular location at the same time. As these sequences are far lower in number and may confuse the machine learning classifier while predicting circRNAs’ sub-cellular locations, we have discarded these sequences in order to obtain a collection of 15,807 sequences that solely belong to one sub-cellular location at a time. We eliminate overlapping sequences of different tissues to obtain a clean dataset of 15,553 sequences annotated against 8 sub-cellular localities. A total of 14,780 sequences are found in Exosome, 342 in Cytosol, 203 in Nucleus, 109 in Membrane, 35 in Nucleolus, 30 in Chromatin, 30 in Nucleoplasm, and 24 in Insoluble Ctytoplasm. Further, in order to eliminate redundant sequences, we leverage CD-HIT2D [101] tool. A number of sequences from each sub-cellular location that are found to be highly similar to the sequences of other sub-cellular locations are excluded. For all sub-cellular locations, a stringent similarity threshold of 0.8 is used following the published literature [102,103]. Finally, we obtain a benchmark dataset of 1,205 sequences in which we manage to retain 482 sequences of Exosome, 319 of Cytosol, 191 of Nucleus, 100 of Membrane, 32 of Nucleolus, 32 of Nucleoplasm, 27 of Chromatin, and 22 of Insoluble Cytoplasm. Statistics of the benchmark dataset along with the entire workflow used to collect and process the benchmark dataset are given in Figure 8.

Although the backsplicing junction of a sequence is specific for circRNA and different from linear mRNA, we have not distinguished common sequences between circRNAs and their host mRNAs and used full circular RNA sequences for training the predictive model. This is primarily due to fact that we have used circular RNA sequences that have been experimentally identified in different subcellular compartments and the prime objective of this study is to complement wet-lab experiments for accurate detection and validation of circular RNA subcellular localizations through the development of robust Artificial Intelligence-based methods.

### 4.3. Evaluation Metrics

In order to evaluate the performance of computational methodologies, an appropriate selection of evaluation measures is really crucial to draw certain conclusions. Considering the effectiveness and wide adoption of accuracy (ACC), specificity (SP), F1-score, matthews correlation coefficient (MCC), and area under the receiver operating characteristics (AUC-ROC) [104], we evaluate the performance of proposed Circ-LocNet in terms of these 5 evaluation metrics. A brief description accompanied with mathematical expression for each selected evaluation metric is provided below:

(11) $$f\left(x\right)=\left\{\begin{array}{cc} \mathrm{Accuracy}\;\left(\mathrm{ACC}\right)=\left(O_{-}^{+}+O_{+}^{-}\right)/\left(O^{+}+O^{-}\right) & 0\leq ACC\leq 1\\ \mathrm{Specificity}\;\left(\mathrm{SP}\right)=\left(O_{+}^{-}/\left(O_{+}^{-}+F_{-}^{+}\right)\right) & 0\leq SP\leq 1\\ \mathrm{MCC}=\frac{\left(\left(O_{-}^{+}*O_{+}^{-}\right)-\left(F_{-}^{+}*F_{+}^{-}\right)\right)}{\left(O_{-}^{+}+F_{-}^{+}\right)*\left(O_{-}^{+}+F_{+}^{-}\right)*\left(O_{+}^{-}+F_{-}^{+}\right)*\left(O_{+}^{-}+F_{+}^{-}\right)} & -1\leq MCC\leq 1\\ F1-\mathrm{score}=2*\frac{\left[Precision*Recall\right]}{\left[Precision+Recall\right]} & 0\leq F1-score\leq 1 \end{array}\right.$$

In Equation (11), O^+^ refers to true positives and false positives whereas O^−^ represents the true negatives and false negatives. The number of positive class sequences which are correctly predicted as positive are represented as $O_{-}^{+}$ and number of negative class sequences which are accurately predicted as negative are represented as $O_{+}^{-}$. Sequences wrongly predicted into positive class (false positives) are shown as $F_{-}^{+}$ and sequences inaccurately classified into negative class (false negatives) are represented as $F_{+}^{-}$.

Accuracy (ACC) refers to the proportion of instances that have been correctly predicted by the classifier out of all instances. Accuracy usually proves misleading for datasets having imbalanced class distribution, while specificity measures the true negative rate, and recall measures true positive rate. Precision indicates up to what percent of positive identifications are actually correct out of all positive predicted instances. F1-score computes a harmonic mean of precision and recall. All three evaluation metrics (Recall, Precision, F1) are asymmetric as they do not take true negative into account and are greatly influenced by the magnitude of positive class. MCC, on the other hand, takes all 4 entries of the confusion matrix (TP, FP, TN, FN) into account and a high value of MCC indicates that classifier is identifying all corpus classes quite well even if a certain class is disproportionately over- or under-represented.

Trivial evaluation metrics compute the classifier performance by making a comparison between actual and predicted sub-cellular localizations. However, receiver operating characteristic curve (ROC) reveals the performance of the classifier at different thresholds by taking actual labels and predicted label probabilities into account. Traditional evaluation metrics such as accuracy only manage to indicate the actual classifier performance when the dataset is highly balanced, which exposes its bias towards class size. Nevertheless, area under the receiver operating characteristics curve (AU-ROC) accurately determines the classifier performance without being effected by the size of corpus classes, indicating that AU-ROC is neither inclined towards positive class nor negative class.

### 4.4. Experimental Setup

Proposed computational framework Circ-LocNet is implemented using an open-source machine learning library called Scikit-Learn [105]. In order to perform a fair evaluation of the proposed CircLoc-Net on benchmark circRNA sub-cellular localization sequencing dataset, 10-fold cross validation is performed. In K-fold cross validation, the benchmark dataset is equally segregated into *K* subsets, where K−1 subsets are used to train the model and the one leftover subset is utilized to test the model. The entire process is repeated k-times, and in this manner all subsets of the dataset are once used for testing. Final performance is computed by taking the average of performance figures produced by all K-testing experiments. K-fold cross validation elucidates overall performance by eliminating the biaseness which a classifier may have towards the split of the dataset.

Taking the effectiveness of grid search for automated parameter search [106] into account, we use grid search to determine the optimal values of diverse hyperparameters related to sequence encoding and the generalizeability of machine learning classifiers. Inspired by the studies of Le et al. [107] and Asim et al. [108], experimentation is performed by varying the residues parameter *k* from 2 to 5. Residue-encoding specific parameters such as K-gap initial range is defined as 2 to 5 following the state-of-the-art sequence representation learning toolkits such as iLearnPlus [49]. Turning towards machine learning classifiers, tree-based classifiers are evaluated using both gini and entropy criterion where the estimator range is varied from 20 to 200, dicriminative classifier “SVM” is evaluated using linear, polynomial, and radial basis kernel, and generative classifier Naive Bayes smoothing ranges falls between 1e–1 to 1e–9.

## 5. Conclusions

This study achieves a number of milestones regarding sub-cellular localization of circular RNAs by performing a pioneering work using Artificial Intelligence. Circ-LocNet identifies important sequence features which largely influence the generalizeability of diverse sub-cellular localization predictors. Using only 1K training samples, Circ-LocNet achieves a peak AU-ROC score of 90%, accuracy of 69%, and F1-score of 63% solely using residue frequency information and a precisely deep decision tree-based classifier. With the current preciseness, time efficiency, and effectiveness of the predictive pipeline, we consider that Circ-LocNet can achieve consistent and production-ready performance as well by just increasing the training data. A compelling future line of current work would be to investigate whether over-sampling and under-sampling approaches are appropriate and good enough to further raise the performance of Circ-LocNet.

## Figures and Tables

**Table 1:** Accuracy Produced by 5 Different Machine Learning Classifiers using 7 Distinct Sequence Descriptors.

Sequence Descriptors	RandomForest	Xgboost	Naive Bayes	SVM	Adaboost
EIIP	0.675	0.663	0.092	0.403	0.523
zCurve	0.621	0.612	0.270	0.405	0.526
triMonoKGap	0.688	0.678	0.225	0.463	0.559
diMonoKGap	0.643	0.665	0.214	0.405	0.557
Kmer	0.685	0.670	0.606	0.469	0.490
RCkmer	0.676	0.651	0.591	0.489	0.521
pseudoKNC	0.685	0.658	0.249	0.446	0.564

**Table 2:** F1-score Produced by 5 Different Machine Learning Classifiers using 7 Distinct Sequence Descriptors.

Sequence Descriptor	RandomForest	Xgboost	Naive Bayes	SVM	Adaboost
EIIP	0.619	0.613	0.109	0.232	0.532
zCurve	0.585	0.585	0.304	0.236	0.525
triMonoKGap	0.634	0.635	0.260	0.334	0.561
diMonoKGap	0.602	0.624	0.248	0.235	0.562
Kmer	0.623	0.622	0.589	0.351	0.493
RCkmer	0.617	0.601	0.582	0.385	0.521
pseudoKNC	0.630	0.613	0.282	0.308	0.565

**Table 3:** Best Performing K-Order Sequence Descriptor Fusions across 5 Different Machine Learning Classifiers.

Machine Learning Classifier	Best Performing K-Order Sequence Descriptor Fusion					
	**2nd Order**	**3rd Order**	**4th Order**	**5th Order**	**6th Order**	**7th Order**
Random Forest	TriMonoKGap+ PseudoKNC	RCKmer+ zCurve+ Kmer	diMonoKGap+ RCKmer+ triMonoKGap+ pseudoKNC	diMonoKGap+ RCKmer+ triMonoKGap+ pseudoKNC+ zCurve	diMonoKGap+ EIIP+ triMonoKGap+ pseudoKNC+ zCurve+Kmer	diMonoKGap, RCKmer, EIIP+ triMonoKGap+pseudoKNC+ zCurve+Kmer
Xgboost	Kmer+ diMonoKGap	pseudoKNC+ diMonoKGap+ RCKmer	Kmer, triMonoKGap+ zCurve+ RCKmer	Kmer+triMonoKGap+ zCurve+ diMonoKGap+ RCKmer	pseudoKNC+triMonoKGap+ EIIP+zCurve+ diMonoKGap+RCKmer	diMonoKGap+EIIP+RCKmer+ triMonoKGap+pseudoKNC + zCurve+Kmer
Naive Bayes	RCKmer+ Kmer	RCKmer+Kmer +pseudoKNC	RCKmer+pseudoKNC+ ZCurve+Kmer	diMonoKGap+RCKmer+ pseudoKNC+ zCurve+Kmer	diMonoKGap+RCKmer+ triMonoKGap+pseudoKNC +zCurve+Kmer	diMonoKGap+EIIP+RCKmer+ triMonoKGap+pseudoKNC + zCurve+Kmer
SVM	diMonoKGap+ EIIP	diMonoKGap+EIIP+ zCurve	diMonoKGap+ EIIP+RCKmer +triMonoKGap	diMonoKGap+EIIP+RCKmer +triMonoKGap+pseudoKNC	diMonoKGap+EIIP+ RCKmer+triMonoKGap+ pseudoKNC+zCurve	diMonoKGap+EIIP+RCKmer+ triMonoKGap+pseudoKNC + zCurve+Kmer
AdaBoost	diMonoKGap+ pseudoKNC	diMonoKGap+ pseudoKNC +zCurve	diMonoKGap+RCKmer+ triMonoKGap+zCurve	diMonoKGap+RCKmer+ pseudoKNC+ zCurve+Kmer	diMonoKGap+RCKmer+ triMonoKGap+pseudoKNC +zCurve+Kmer	diMonoKGap+EIIP+RCKmer+ triMonoKGap+pseudoKNC + zCurve+Kmer

**Table 4:** Accuracy Produced by Top Performing K-Order Sequence Descriptor Fusions Across 5 Different Machine Learning Classifiers.

Encoder Fusion	RandomForest	Xgboost	Naive Bayes	SVM	Adaboost
2nd-order	0.695	0.689	0.605	0.681	0.564
3rd-order	0.693	0.689	0.249	0.683	0.576
4th-order	0.694	0.688	0.249	0.682	0.571
5th-order	0.687	0.686	0.247	0.681	0.562
6th-order	0.692	0.684	0.239	0.674	0.551
7th-order	0.683	0.678	0.221	0.673	0.531

**Table 5:** F1-score Produced by Top Performing K-Order Sequence Descriptor Fusions Across 5 Different Machine Learning Classifiers.

Encoder Fusion	RandomForest	Xgboost	Naive Bayes	SVM	Adaboost
2nd-order	0.643	0.637	0.587	0.621	0.566
3rd-order	0.632	0.641	0.282	0.624	0.575
4th-order	0.641	0.638	0.282	0.622	0.571
5th-order	0.633	0.637	0.278	0.621	0.564
6th-order	0.637	0.634	0.271	0.613	0.551
7th-order	0.634	0.629	0.251	0.613	0.531

## Data Availability

Circ-LocNet is deployed as a very first circRNAs sub-cellular localization prediction platform at https://circ_rna_location_predictor.opendfki.de/.
